# Case Report: Methylphenidate and venlafaxine improved abdominal nociplastic pain in an adult patient with attention deficit hyperactivity disorder, autism spectrum disorder, and comorbid major depression

**DOI:** 10.3389/fpain.2024.1394131

**Published:** 2024-08-21

**Authors:** Satoshi Kasahara, Miwako Takahashi, Kaori Takahashi, Taito Morita, Ko Matsudaira, Naoko Sato, Toshimitsu Momose, Shin-Ichi Niwa, Kanji Uchida

**Affiliations:** ^1^ Department of Anesthesiology and Pain Relief Center, The University of Tokyo Hospital, Tokyo, Japan; ^2^ Department of Pain Medicine, Fukushima Medical University School of Medicine, Fukushima, Japan; ^3^ Institute for Quantum Medical Science, National Institutes for Quantum Science and Technology, Chiba, Japan; ^4^ Department of Dental Anesthesiology, Tokyo Dental College, Tokyo, Japan; ^5^ Nursing Department, The University of Tokyo Hospital, Tokyo, Japan; ^6^ Institute of Engineering Innovation, School of Engineering, The University of Tokyo, Tokyo, Japan; ^7^ Department of Psychiatry, Aizu Medical Center, Fukushima Medical University, Fukushima, Japan

**Keywords:** abdominal nociplastic pain, central sensitization, attention deficit hyperactivity disorder, autism spectrum disorder, depression, methylphenidate, venlafaxine, single-photon emission computed tomography

## Abstract

**Introduction:**

Nociplastic pain (NP), classified as a third type of pain alongside nociceptive and neuropathic pain, is chronic pain arising from the amplification of nociceptive stimuli through central sensitization, despite the absence of tissue damage, sensory nerve damage, or disease. An important clinical feature of NP is that it is not only associated with pain but also with sensory hypersensitivity to sound and light and cognitive dysfunction, including mood and attention disorders. Recent studies have suggested that depression and developmental disorders, such as attention deficit hyperactivity disorder (ADHD) and autism spectrum disorder (ASD), coexist with NP at high frequency. Additionally, cognitive impairment in individuals with NP may be associated with these psychiatric comorbidities. However, to our knowledge, there are no reports on (1) multidimensional evaluation and diagnostic details of abdominal NP in adults with ADHD/ASD; (2) how ADHD drugs and antidepressants are administered when ADHD and depression coexist with NP; and (3) how central sensitization, brain function, and family relationship problems underlying NP are altered by treatments of ADHD and depression.

**Case presentation:**

Herein, we present the case of a 51-year-old woman with abdominal NP. She developed severe right lower abdominal pain and underwent a thorough medical examination; however, the physical, medical cause remained unknown, making treatment challenging. Additionally, she took time off work as she began to complain of insomnia and anxiety. She was referred to our pain center, where a diagnosis of depression, ADHD, and ASD was confirmed, and treatment with ADHD medication was initiated. While ADHD medications alone did not yield sufficient improvement, a combination of methylphenidate and the antidepressant venlafaxine eventually led to improvements in abdominal NP, depression, ADHD symptoms, central sensitization, and family relationship issues. During treatment, cerebral blood flow in the anterior cingulate, prefrontal, and parietal cortices also improved.

**Conclusion:**

The treatment of comorbid depression is important while treating NP, and venlafaxine may be effective, especially in cases of comorbid ADHD/ASD. Screening for developmental disorders and depression is required in patients with abdominal NP.

## 1 Introduction

Recently, the International Association for the Study of Pain defined chronic pain as “nociplastic pain (NP), which arises from altered nociception despite no clear evidence of actual or threatened tissue damage causing the activation of peripheral nociceptors or evidence of disease or lesion of the somatosensory system causing the pain” (1). NP is a mechanism-based classification of chronic pain and is the third pain type alongside nociceptive pain and neuropathic pain. It pertains to pain that has traditionally been treated as psychogenic pain or a somatoform disorder (1, 2). The underlying mechanism is thought to involve plastic changes in the central neural circuits that transmit nociception, causing central sensitization that amplifies external stimuli (3). An important NP clinical feature is that pain rarely occurs alone and is accompanied by cognitive dysfunction. NP is diagnosed by the exclusion of nociceptive and neuropathic pain and the presence of central nervous system symptoms (hyperalgesia or fatigue, sensory sensitivity to sound or light, sleep disturbances, mood disorders, or cognitive dysfunction in focusing attention or memory) (3). In the context of mood disorders, addressing comorbid depression in patients with NP is crucial, as depression coexists in 40%–85% of patients with chronic pain, including NP. This increases medical visits and costs, making treatment difficult (4, 5).

Recently, attention deficit hyperactivity disorder (ADHD) and autism spectrum disorder (ASD) have often been observed to coexist with fibromyalgia (6–8), chronic low back pain (9–12), and idiopathic oral–facial pain (13–15), all of which are representative disorders of NP. This suggests that ADHD and ASD may contribute to the development of NP and its associated cognitive dysfunction, including attention disorders and sensory hyperactivity (16). Furthermore, ADHD medication can improve both pain and cognitive dysfunction in individuals with comorbid NP and ADHD (13, 17, 18). This is a promising new approach for NP, which is often refractory to treatment.

In addition to the aforementioned fibromyalgia, chronic low back pain, and idiopathic oral–facial pain, chronic abdominal pain is also one of the NP syndromes (2). According to a longitudinal study by Asztély and colleagues, 77% of female patients diagnosed with ADHD or ASD in childhood had chronic pain when followed into adulthood, with 30% experiencing chronic abdominal pain, suggesting an association between chronic abdominal pain and ADHD/ASD (19). Furthermore, patients with ADHD who were taking central stimulant medications during adulthood had a significantly lower prevalence of chronic pain compared to those not taking such medications (19). Lipsker et al. reported that approximately 60% of child and adolescent patients with chronic pain reported chronic abdominal pain, with about 20% exhibiting clinical-level ADHD symptoms, 14% showing clinical-level ASD symptoms, and 26% experiencing both ADHD and ASD symptoms (20). However, it is important to note that ADHD/ASD in women with chronic pain is often high-functioning and therefore likely to be overlooked.

Although previous studies by Asztély et al. have demonstrated the preventive effects of central stimulant medications on chronic pain associated with developmental disorders, to our knowledge, there are no detailed case reports on the multifaceted evaluation and diagnosis of abdominal NP in adults with ADHD/ASD, as well as the treatment plan involving central stimulant medications. Moreover, no reports address whether ADHD medications or antidepressants are more effective when ADHD and depression coexist with NP. Furthermore, no reports detail how central sensitization, brain function, and family relationship issues underlying NP are altered by ADHD or antidepressant medications. We report a case in which a combination of the ADHD drug methylphenidate (MP) and the antidepressant venlafaxine (VFX) ultimately improved abdominal NP in an adult patient, accompanied by improvements in ADHD symptoms, central sensitization, cerebral blood flow, and family relationship problems.

## 2 Case description

The patient was a 51-year-old woman (height, 166 cm; weight, 48 kg). Her family and medical history revealed no notable findings. Prior to pain onset, she had no psychiatric diagnoses throughout her childhood to adulthood. Since graduating from college, she had worked as a clerk and lived with her husband, without any children. She did not experience serious menopausal symptoms, and her work and daily lifestyle remained unchanged when the NP started. In April 2019, she was diagnosed with dental caries and its impact on her nerves during a dental checkup at her workplace. Upon discovering that nerve loss can cause premature tooth loss, she extensively researched dental caries online. Overwhelmed by the abundance of information, she ruminated only on the negative aspects and began experiencing anxiety. Seeking a solution, she visited three dental clinics in succession, but her inability to make a decision and understand the information only exacerbated her insomnia, depression, and agitation. She began caries treatment at the fourth dentist's office, but each procedure took over an hour due to her sensory sensitivity. This caused notable shaking of her neck and body when local anesthesia was administered, dental lights were turned on, buccal mucosa and tongue were compressed, and splints were inserted. Despite completing her caries treatment, her depression continued.

In May 2019, lower right abdominal pain began. In June 2019, she had blood tests, magnetic resonance imaging, and a colonoscopy at a gastroenterologist's office to rule out tumor or inflammatory bowel disease as the cause. They diagnosed her with suspected psychogenic abdominal pain due to stress or other factors and determined that further medical treatment was unnecessary. The abdominal pain persisted, and her insomnia worsened. She visited a psychosomatic clinic in August 2019 and received acetaminophen 3,000 mg/day, loxoprofen sodium 180 mg/day, amoxapine 30 mg/day, and duloxetine 40 mg/day, but neither the pain nor the insomnia improved. She was referred to a psychiatrist at the University of Tokyo Hospital Pain Center on December 2, 2019, because the cause of her pain was unknown and difficult to treat. She had insomnia and anxiety and had to take a leave of absence from work.

### 2.1 Diagnostic assessments

Subjective pain intensity was assessed using a numerical rating scale (NRS) (21). Regarding the amount of change in NRS scores for chronic pain, the minimum clinically important difference (MCID) considers a decrease of −2 or more points in the NRS to be either substantial or optimal (22). Health-related aspects of quality of life were assessed using the EuroQoL 5 Dimension (EQ-5D) (23), with EQ-5D scores of 0, 1.0, and 0.08 indicating death, perfect health, and MCID, respectively (24). Anxiety and depression were assessed using the Hospital Anxiety and Depression Scale-Anxiety/Depression (HADS-A/D) (25). A score of 11 or higher on the HADS was considered a clinical level of anxiety or depression (26), and the MCID for the HADS is 1.5 (27). Pain-related catastrophizing thoughts were assessed using the Pain Catastrophizing Scale (PCS) (28). PCS scores of 30 or higher were considered severe, with an MCID of 6.48 points (24). Central sensitization was assessed using the Central Sensitization Inventory-9 (CSI-9) (29). A score of 20 or higher was considered positive for central sensitization (30). Family relationship issues contributing to the maintenance of chronic pain were assessed using the Multidimensional Pain Inventory (MPI) (31, 32). The MPI calculates a dysfunctional (DYS) score, representing the family's tendency toward overprotective behavior reinforcing the patient's pain, and an interpersonally distressed (ID) score, indicating the patient's tendency to feel blamed by the family. Based on the balance of these scores, the patient is categorized as DYS, ID, or adaptive coper (AC). In the DYS category, the patient and family tend to have a symbiotic relationship, and family relationship conflicts are less apparent. In the ID category, the patient feels blamed by the family and is more aware of family relationship conflicts. In the AC type, the patient maintains an appropriate interpersonal distance despite conflicts.

**Figure 1:**
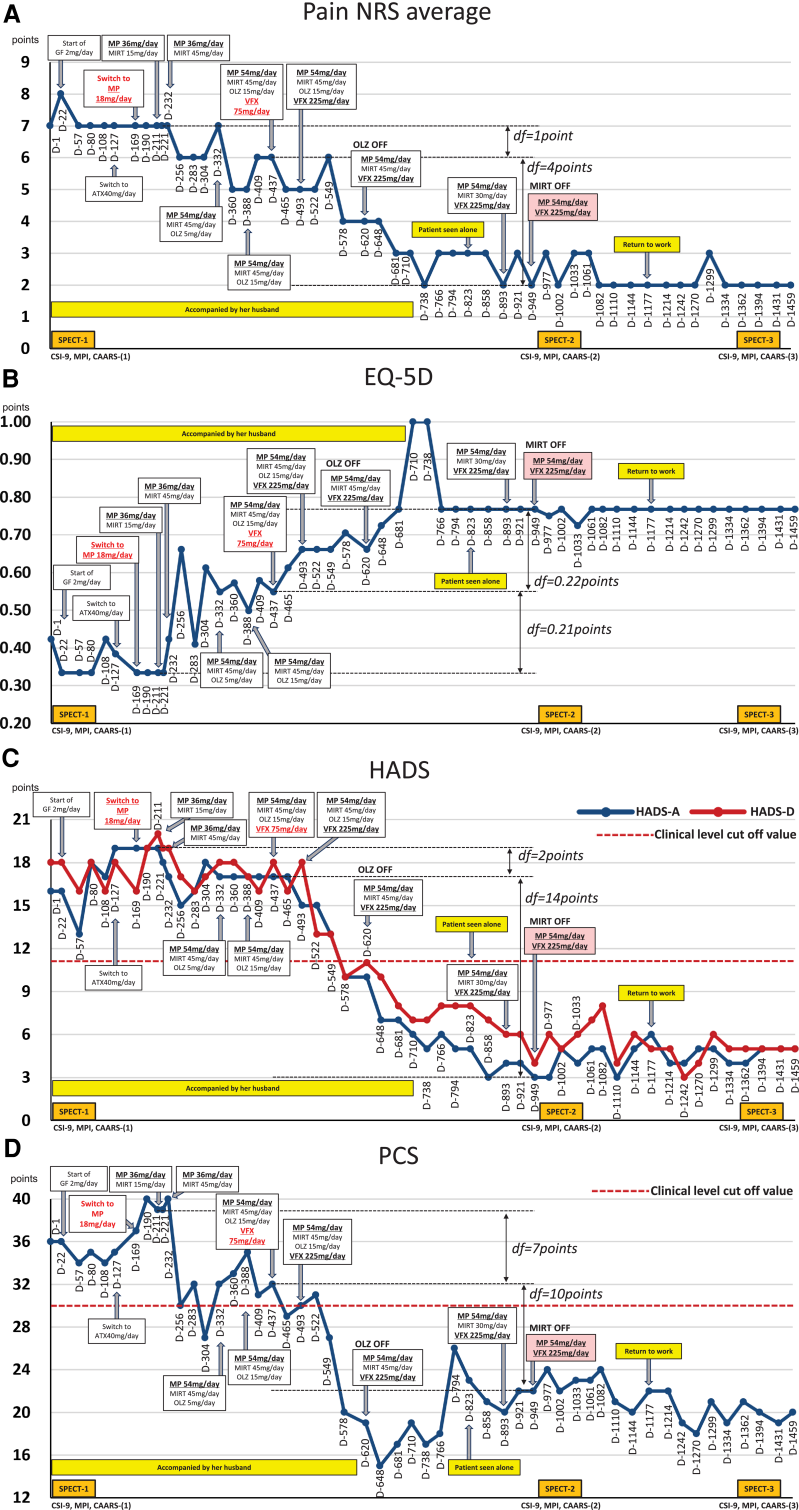
Course of treatment and subjective parameters. Additional MP and VFX interventions that may have contributed to the improvement in the patient's nociplastic pain status are highlighted in red underlined font. Subsequent changes in MP and VFX doses are shown in black bold underlined font. **(A)** Average pain NRS score. **(B)** EQ-5D score. **(C)** HADS score. **(D)** PCS score. ATX, atomoxetine; CAARS, Conners’ adult ADHD rating scale; CSI-9, central sensitization inventory-9; D, day; df, difference; EQ-5D, EuroQoL-5 dimension; GF, guanfacine; HADS-A/D, hospital anxiety and depression scale-anxiety/depression; MIRT, mirtazapine; MP, methylphenidate; MPI, multidimensional pain inventory; NRS, numerical rating scale; OLZ, olanzapine; PCS, pain catastrophizing Scale; SPECT, single-photon emission computed tomography; VFX, venlafaxine.

**Figure 2:**
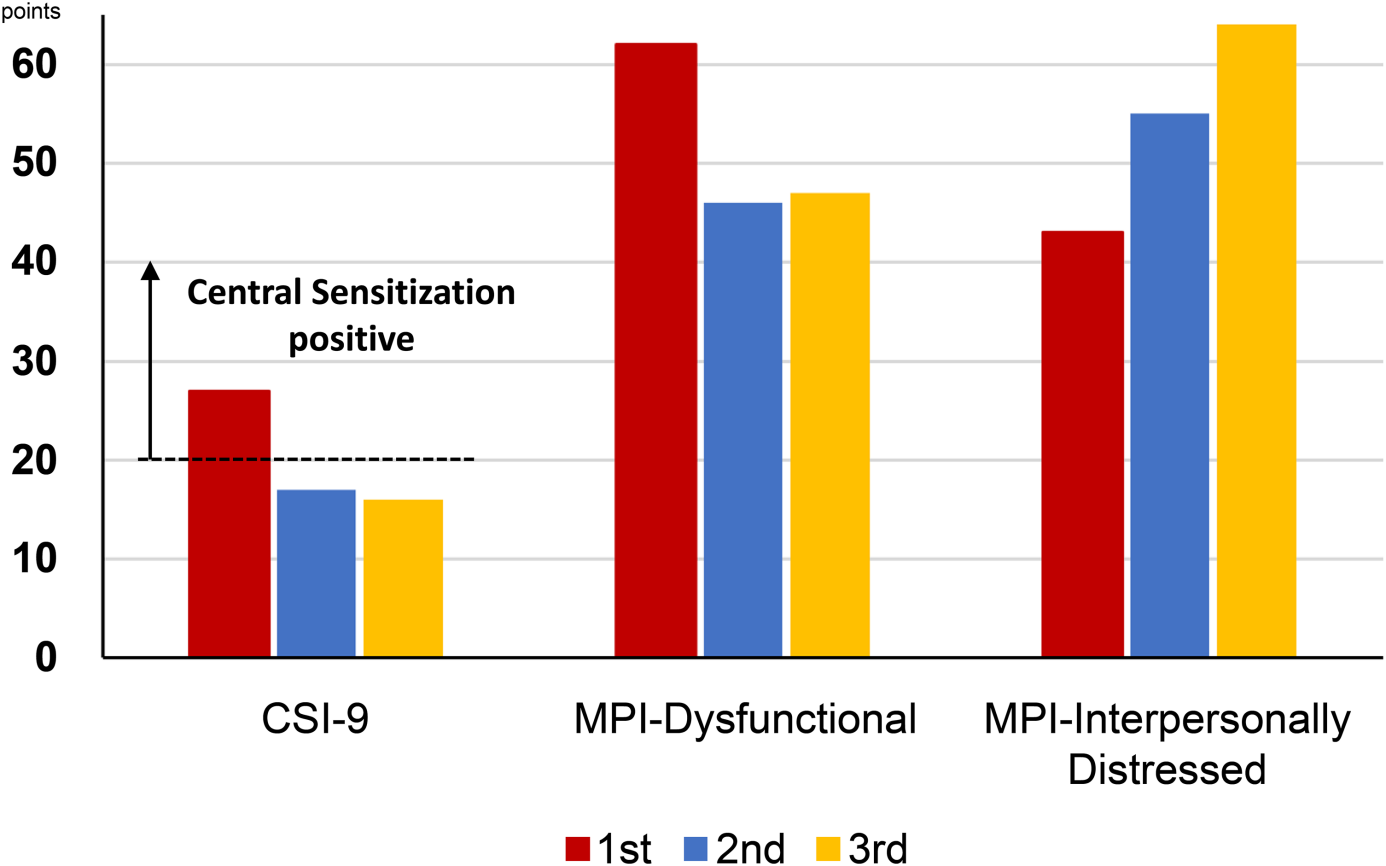
Changes in the CSI-9 and MPI scores along the course of treatment. CSI-9, central sensitization inventory-9; MPI, multidimensional pain inventory.

The average NRS score for abdominal pain at the first visit was 7/10 points; EQ-5D score, 0.423; HADS-A score, 16/21; HADS-D score, 18/21; PCS score, 36/52; CSI-9 score, 27/36; MPI DYS score, 62; and MPI ID score, 43. The classification was DYS type (Figures 1, 2). Her husband, a workaholic, used to listen absentmindedly to the patient's stories and paid little attention to her symptoms. However, when the patient started taking time off work, he became a dedicated caregiver and even accompanied her to the hospital.

Her chief complaint was chronic abdominal pain, but it involved more than just abdominal pain. She also presented with allodynia, dental pain, headaches, and central nervous system complications such as sensitivity to touch, pressure, sound, and light during dental procedures. Additionally, she experienced sleep disturbances, severe fatigue, and poor concentration. These symptoms were reflected in her high score of 27 on the CSI-9. Each of the 9 items was scored as follows: 1. Unrefreshed in the morning: 4/4, 2. Muscles stiff/achy: 1/4, 3. Pain all over the body: 3/4, 4. Headaches: 3/4, 5. Poor sleep: 4/4, 6. Difficulty concentrating: 3/4, 7. Stress worsens symptoms: 3/4, 8. Tension in the neck and shoulders: 4/4, 9. Poor memory: 2/4. This suggests that central sensitization may be a factor in her abdominal pain. Her abdominal pain had no clear trigger, and both nociceptive pain and neuropathic pain were ruled out, confirming that her pain was indeed nociplastic. In other words, her distress was not limited to abdominal pain alone but also included various central nervous system symptoms associated with NP.

SK, a well-experienced psychiatrist, differentiated comorbid psychiatric disorders according to the Diagnostic and Statistical Manual of Mental Disorders, Fifth Edition (DSM-5) (33) criteria. The patient had a persistently depressed mood, decreased interest, insomnia, agitation, fatigue, and impaired thinking and judgment at pain onset, which was classified as major depression. She also spent hours a day searching the Internet for information about her physical symptoms. Thus, she was diagnosed with somatic symptom disorder, without manic episodes, obsessive-compulsive disorder, or psychotic disorders, and no complications or history of acute stress disorder or post-traumatic stress disorder.

She took a long time to answer the questions in the pre-consultation questionnaire because she was particular about the questions' wording. The patient's husband was present during the examination. Her facial expression did not change much, and her speech was noticeably formal and overly awe-inspiring. Additionally, the patient frequently digressed, ignoring SK's prompt to move on to the next patient. Prior to experiencing depression and pain, her interpersonal work relationships were strained, and she frequently made serious mistakes. She carelessly misplaced important documents and often caused displeasure among doctors at previous medical visits. Lastly, she left her belongings behind in the examination room after the consultation.

**Figure 3:**
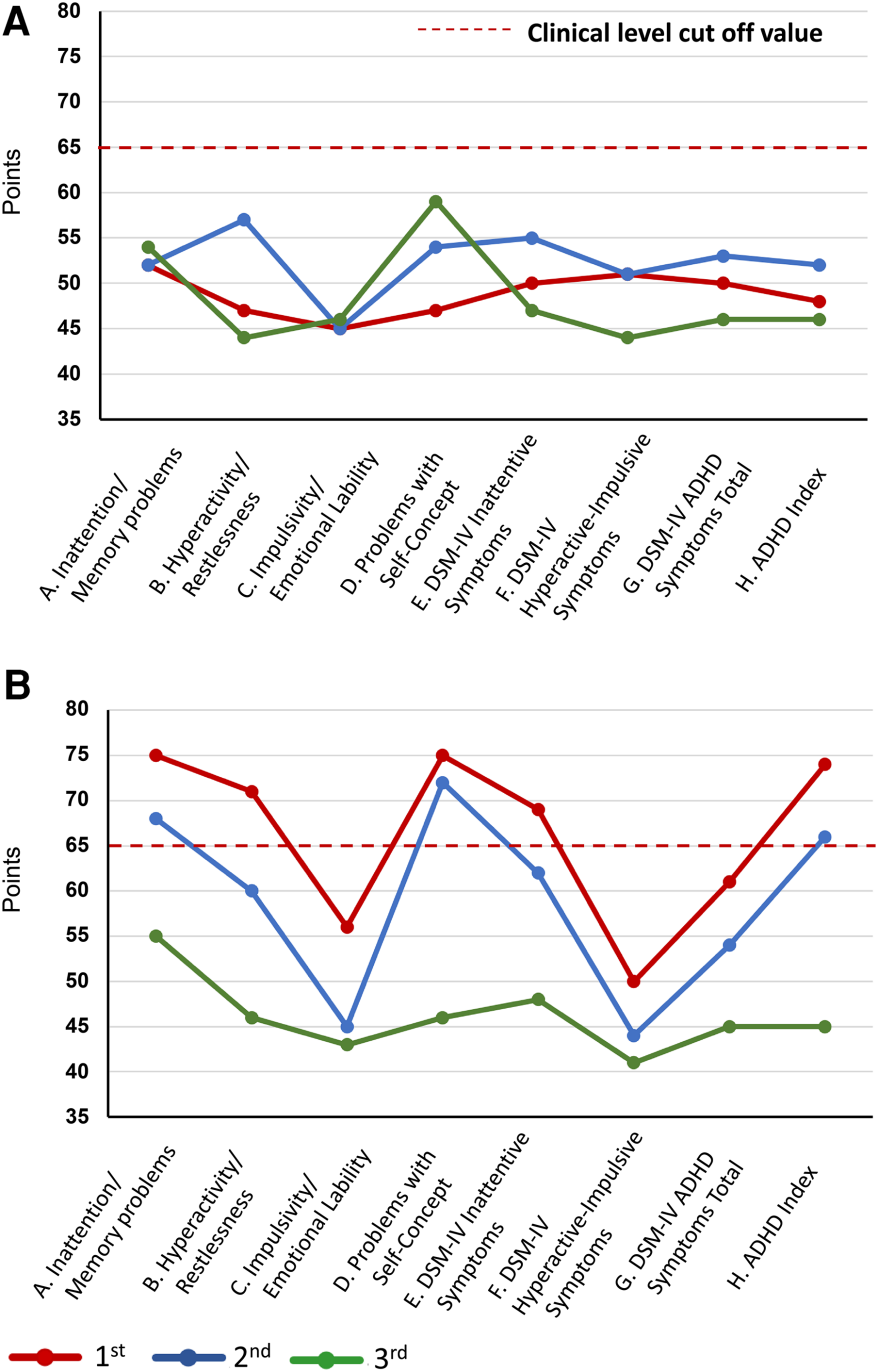
Changes in the CAARS scores during treatment. Changes in the CAARS scores over the course of treatment. **(A)** CAARS-S and **(B)** CAARS-O. ADHD, attention deficit hyperactivity disorder; CAARS-S/O, Conners’ Adult ADHD Rating Scale Self-report/Observer-rated; DSM-IV, Diagnostic and Statistical Manual of Mental Disorders, Fourth Edition.

Since ADHD has been reported to be a frequent comorbidity in NP conditions (17), SK suspected ADHD comorbidity in this patient and performed ADHD symptom assessment and diagnostic confirmation from day 1 to day 22. ADHD symptoms were assessed using the Conners' Adult ADHD Rating Scale (CAARS) self-report (CAARS-S) and observer-rated (CAARS-O) questionnaires (34), completed by the patient and her husband, respectively. The patient's CAARS results showed that her CAARS-O subscale score was >65, and her ADHD symptoms were rated at the clinical psychiatric level by her husband (Figure 3). The CAARS manual states that if the results of the CAARS-S and CAARS-O differ, it may indicate that either the patient or the observer is denying or is not fully aware of the patient's ADHD symptoms (34). Since this patient was able to describe episodes related to ADHD symptoms when interviewed in detail, it is believed that she was not denying her ADHD symptoms but was not fully aware of their extent. From an early age, she was identified as a highly active individual who had difficulty sitting still and had a tendency to forget things quickly. As a child, she was very shy and reluctant to speak in public. In adulthood, she met 8/9 of the DSM-5 ADHD diagnostic criteria for inattention and 8/9 for hyperactivity-impulsivity, resulting in a combined-type ADHD diagnosis.

Regarding persistent deficits in social communication and social interactions according to the DSM-5 ASD, the following items applied. She often angered doctors by asking questions unilaterally of interest to her beyond her consultation time or by repeatedly calling the outpatient clinic to ask questions, disregarding that the doctors were busy examining other patients. This was categorized as a “deficit in social-emotional reciprocity.” She showed little change in facial expression and a monotone tone of voice when speaking during the examination, considered as “deficits in nonverbal communicative behaviors used for social interaction.” Throughout her childhood and adulthood, she was easily taken in by jokes, was often straightforward, had difficulty in joining a circle of friends, and was often teased and bullied. She was warned by a nurse for using the nurses' desk at the nurses’ station without permission while waiting for an examination. These events indicate “deficits in developing, maintaining, and understanding relationships.”

Regarding restricted, repetitive patterns of behavior, interests, or activities according to the DSM-5 ASD, the following items applied. During the examination, her conversation was often formal and overly polite, and she exhibited a “ritualized pattern of verbal behavior.” She exhibited “highly restricted, fixated interests that are abnormal in intensity or focus,” spending hours on the Internet researching the negative effects of dental caries on nerves and its treatment, resulting in depression. Since childhood, she has had a hypersensitivity to tags attached to clothing lapels and has always removed these tags. She also exhibited strong sensory sensitivity to pain, touch, and light, which made dental treatment difficult. These characteristics were considered as “hypersensitivity to sensory input.” Based on these observations, the behavioral features of ASD were consistently present throughout her childhood and adulthood, and she was diagnosed with ASD according to the DSM-5.

Based on the course of this case and the results of the evaluation and diagnosis, the following clinical conditions were inferred. First, the patient exhibited ADHD and ASD characteristics since childhood, i.e., before the onset of abdominal NP and depression. These characteristics tended to cause problems in interpersonal relationships and work. However, as mentioned in the introduction, she had high-functioning ADHD/ASD. As a result, she was able to graduate from college and work as an office worker. Her developmental disability went undiagnosed from childhood and adolescence into adulthood. Later, when her caries was pointed out to her, she immersed herself in researching its treatment. However, she became confused, unable to organize and discard the large amount of information she had gathered. She also fixated excessively on the negative information. These manifestations were thought to be related to her ADHD/ASD cognitive dysfunctions, such as “executive functioning,” “excessive concentration,” and “limited interest.” These dysfunctions ultimately led to her developing depression and experiencing abdominal NP. In addition, the high CSI-9 score suggested an amplification of nociceptive transmission in the central nervous system. However, the cognitive dysfunctions associated with her NP, such as sensory hypersensitivity and inattention, were present before the onset of abdominal NP and could also be attributed to her innate ADHD/ASD. In other words, ADHD/ASD, depression, and central sensitization were thought to be involved in the onset of her NP. Furthermore, when she began taking a leave of absence, her husband, who had previously been indifferent to her, became overprotective. This change in his behavior was also believed to have contributed to the maintenance of her NP.

### 2.2 Therapeutic interventions and outcomes

**Figure 4:**
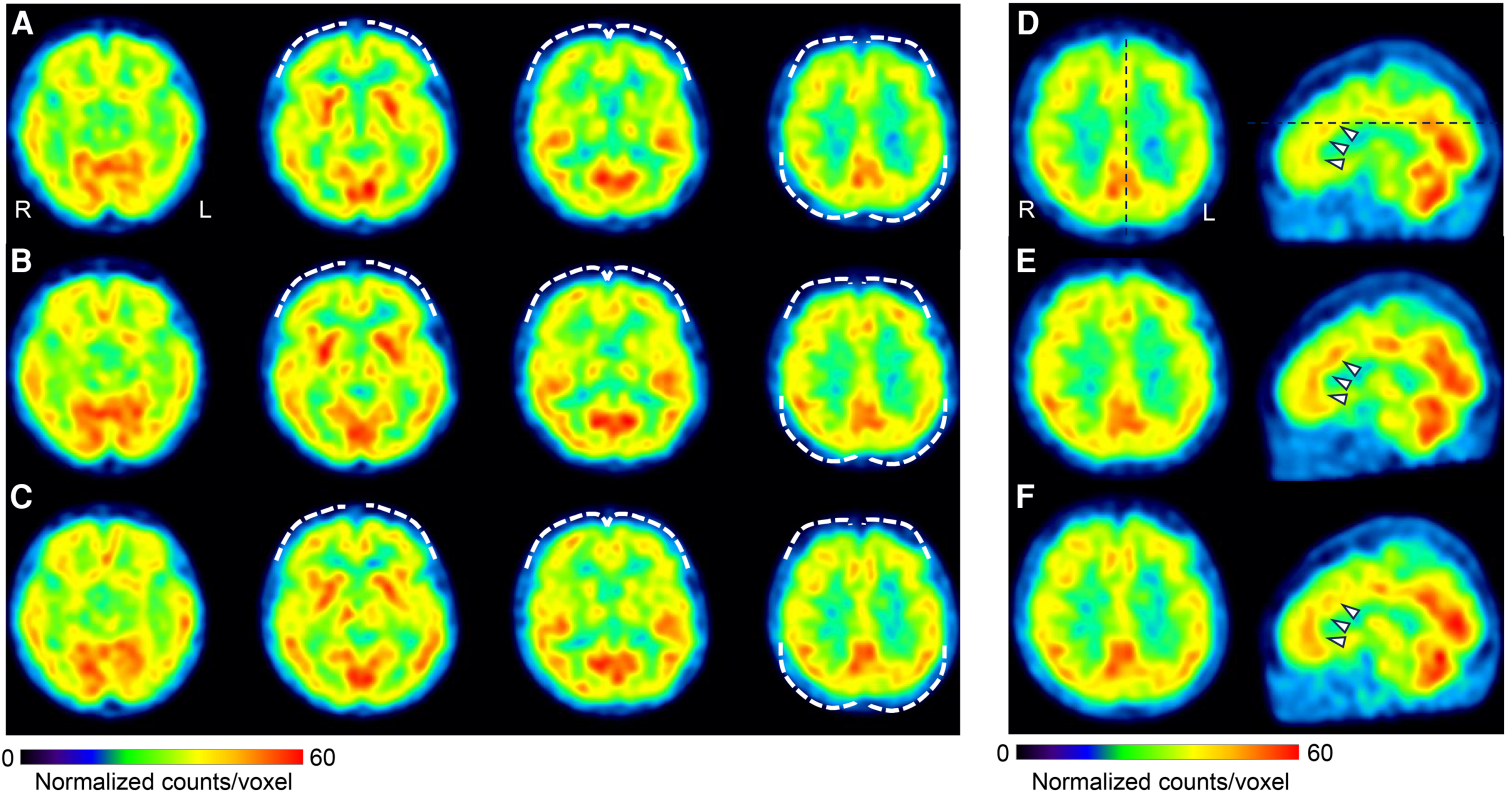
Changes in cerebral blood flow during treatment. Brain perfusion SPECT images. The figure shows four axial images from left to right: basal frontal to upper frontoparietal levels **(A–C)**, and axial and sagittal images of the anterior to middle cingulate cortices level **(D–F)**. The SPECT images were acquired at three different time points: before treatment initiation **(A,D)**, day 1,002 **(B,E)**, and day 1,394 **(C,F)**. The voxel values were normalized using the average counts per voxel, with the cerebellum set at 50. The color bar represents a range from 0 to 60. Mild hypoperfusion of the bilateral prefrontal and lateral parietal cortices **(A)** improved slightly in **(B)** and was maintained in **(C)** (white dashed curves). The mean normalized voxel values for each image from **(A)** to **(C)** are 38.7, 40.0, and 39.4 in the frontal lobe, and 40.9, 42.5, and 43.0 in the lateral parietal cortices. The anterior and middle cingulate cortices are identified in **(D)** (arrows), and the blood flow was increased in **(E)** and **(F)** The mean normalized voxel values for each image from **(D)** to **(F)** are 43.6, 45.1, and 45.8 in the anterior and middle cingulate cortices. Voxel value normalization and calculations were performed using PMOD, version 3.7 (PMOD Technologies Ltd., Zurich, Switzerland). SPECT, single-photon emission computed tomography.

On day 22, she reported abdominal pain and several other symptoms including toothache, headache, sweating due to heating, numbness in her upper limbs and face, insomnia, and anxiety. These symptoms affected the examination results. Before treatment, she underwent single-photon emission computed tomography (SPECT) which showed mild hypoperfusion in the bilateral prefrontal and lateral parietal cortices (Figure 4). Neither the ADHD drug guanfacine (GF), initiated on day 22, nor the ADHD drug atomoxetine (ATX), switched from GF on day 127, could be continued due to adverse effects. Consequently, she was switched to the ADHD drug MP (18 mg/day) on day 169. Her husband reported that with MP initiation, her abdominal pain complaints decreased, her energy and thinking improved, and she resumed shopping and cooking after several months. She no longer complained of various symptoms in the examination room, and the focus of the conversation became more directed towards abdominal pain.

However, the MP dose was increased to 36 mg/day on day 190 because of increased pain and anxiety during the evening and morning hours, when the MP effects diminished. She and her husband experienced unprecedented improvement with MP, and now they strongly desire medication adjustment to assist her in managing her insomnia, anxiety, and depression. On day 211, when mirtazapine (MIRT) 15 mg/day was added due to severe insomnia complaints, the insomnia improved. On day 232, an increase in MIRT dose to 45 mg/day resulted in increased mental calm and improvement in abdominal pain. On day 256, aripiprazole was administered as augmentation therapy for antidepressants, and on day 283, risperidone was administered; however, both treatments were interrupted by akathisia. On day 332, the MP dose was increased to 54 mg/day, and the atypical antipsychotic olanzapine (OLZ) 5 mg/day was added for insomnia and titrated up to 15 mg/day by day 388.

On day 437, VFX 75 mg/day was added to further improve anxiety and insomnia, and the patient's symptoms improved. Therefore, VFX was titrated to 225 mg by day 493. Pain NRS, HADS, PCS, and EQ-5D scores improved significantly, and OLZ (15 mg/day) was discontinued on day 620. Since day 823, she has been seen alone, unaccompanied by her husband. Because she complained of weight gain, the MIRT dose was reduced to 30 mg/day on day 893, tapered to 15 mg/day, and discontinued on day 949. At this point, all measures—pain NRS, EQ-5D, HADS, and PCS—showed improvement over the MCID.

Subsequently, attempts were made to reduce the MP and VFX doses at the patient's request, but both were continued as the pain and insomnia worsened. On day 1,002, a second CSI-9, MPI, CAARS, and SPECT scan were performed. Pain NRS, EQ-5D, HADS, PCS, and CSI-9 scores normalized; MPI judgment changed to ID type; and SPECT showed a slight increase in blood flow in the prefrontal and parietal cortices and anterior cingulate cortex. On day 1,177, she returned to work for the first time in 3 years, and her condition stabilized. On day 1,394, a third CSI-9, MPI, CAARS, and SPECT scan were performed. The MPI ID score further increased, judgment remained the ID type, the CAARS-O score normalized, and improvement in cerebral blood flow was maintained.

## 3 Discussion

To the best of our knowledge, this case is the first to report the following: (1) details of the evaluation, diagnosis, and course of treatment in abdominal NP with ADHD, ASD, and depression in adulthood; (2) the importance of improving depression with antidepressants, even in cases of chronic pain with ADHD and ASD coexistence; (3) the beneficial effects of MP and pharmacotherapy for depression not only on NP and depression but also on family relationships, aiding in patients' reintegration into society; and (4) the assumed treatment effect of improvement in brain function, reflected in cerebral blood flow and the associated reduction in central sensitization.

This report shows that ADHD and ASD can coexist with nociplastic abdominal pain in adults. Studies on chronic pain and its association with ADHD/ASD include reports on children and adolescents (11, 16, 19, 20, 35) and a case report on atypical odontalgia in an adult (14).

Previous studies have suggested that individuals with ADHD and ASD are more likely to develop chronic pain because of central sensitization and sensory hypersensitivity (36, 37). ADHD and ASD characteristics can predict pain intensity, quality of life, and depression in individuals with chronic pain and are risk factors for severe chronic pain. Therefore, routine screening for developmental disorders is required in patients with NP (16).

Pain and cognitive dysfunction in patients with NP and comorbid ADHD have been shown to improve with ADHD medications (11–15, 17, 18, 35, 38–41), making ADHD identification particularly important in NP practice. However, 80% of adult ADHD cases are missed in psychiatric practice (42), and the diagnosis is more likely to be missed in cases of abdominal NP, which is often managed by internists and pain clinicians. Therefore, aggressive ADHD screening is important in adults with abdominal NP.

However, since the DSM-5 states that “the symptoms are not better explained by another mental disorder,” we must consider the possibility that NP affected attention and executive functioning, resulting in ADHD-like and ASD-like behaviors. However, this patient exhibited characteristics of developmental disabilities even before pain onset, such as during childhood. It is possible that pain did not cause ADHD and ASD but rather amplified pain-induced developmental symptoms (43). This amplification may have made the characteristics of ADHD and ASD more apparent, which could have played a role in confirming the patient's developmental disorder diagnosis. Presumably, the patient had high-functioning developmental disorders that did not necessitate a psychiatric consultation prior to the onset of pain.

Improving depression with antidepressants is crucial, even in cases of chronic pain with comorbid ADHD and ASD. Tricyclic antidepressants and duloxetine are recommended for treating chronic pain and associated depression (44, 45). Switching to MIRT (46) or the combination use of OLZ (45) has also been effective for treatment-resistant depression (TRD). The efficacy of MIRT in combination with serotonin and noradrenaline reuptake inhibitors, such as VFX, and selective serotonin reuptake inhibitors has also been demonstrated for TRD (47). Noradrenaline reuptake inhibition is an important antidepressant mechanism for chronic pain, and VFX efficacy for neuropathic pain and NP has been reported (48, 49).

Additionally, this patient was treated with tricyclic antidepressants and duloxetine for 8 weeks each by her previous doctor but was considered to have TRD, as she did not respond to either pain or depression. It has been reported that in individuals with chronic pain and comorbid ADHD, ADHD medications improve both pain and depression (11–15, 18). However, in our case, GF and ATX were ineffective, and MP did not lead to a significant improvement in all self-administered measures, including depression. Nonetheless, objective pain and behavior improved, as seen by the husband during the time MP was effective. However, adding MIRT to MP in combination with OLZ resulted in significant improvements, and adding VFX resulted in even greater improvements exceeding the MCID for all subjective measures, including pain NRS, EQ-5D, HADS, and PCS.

Combinations of MP and antidepressants, such as VFX, have been shown to be effective for treating TRD (50). Additionally, this combination has been found to be effective in managing ADHD and its associated depression (51). The efficacy of combining MP and an antidepressant like VFX in this case is consistent with previous reports. Furthermore, it has also been found to improve comorbid ADHD and TRD when accompanied by NP. Furthermore, VFX can improve ADHD and ASD core symptoms (52). Therefore, VFX may be considered as a treatment option for individuals with NP with ADHD and ASD characteristics and depression, as observed in this patient.

MP and pharmacotherapy for depression not only improved NP and depression but also improved family relationships and enabled the patient to reintegrate into society. At the initial visit, the patient was classified as having the DYS type on the MPI. Her husband remained in the examination room for a period after treatment initiation, indicating potential reinforcement of the patient's pain behavior through operant conditioning due to the husband's overprotective response. According to MPI theory, educating DYS-type patients and their families about operant treatment is necessary to reduce attention to pain behaviors and reinforce health behaviors (53). From the perspective of behavior function, the patient's pain behavior can be viewed as somatization that serves the purpose of eliciting attention from her workaholic husband. The patient may have been dissatisfied with her husband's indifference, and cognitive therapy and self-assertion training were also deemed necessary to encourage her verbalization of this conflict.

Since SK's pain center lacks a dedicated psychologist and SK's time with each patient is limited to 15–20 min, operant treatment and cognitive therapy could not be implemented in this case. Nevertheless, as the patient's condition improved with MP and medication for depression, the husband was no longer present, and the MPI assessment changed to the ID type. In other words, the improvement in the patient's condition due to pharmacotherapy may have reduced the family's overprotective operant reinforcing response, which is a characteristic of DYS. This improvement could have facilitated improvements in the patient's pain-related symptoms and social life functioning. Operant therapy is crucial for patients with DYS-type chronic pain; however, the shortage of psychologists in clinical settings for patients with chronic pain is an issue (54). Improving family relationships through pharmacotherapy, as demonstrated in this case, is vital.

Improvement in brain function, as reflected in cerebral blood flow and the associated reduction in central sensitization, was assumed to underlie the treatment response in this case. The anterior cingulate and prefrontal cortices also have bidirectional fiber contacts with the descending inhibitory system of pain and are important areas of the brain for pain control (55). In this case, during treatment, there was an improvement in blood flow in the anterior cingulate and prefrontal cortices and a significant improvement in the CSI-9 scores. It is likely that pharmacotherapy in this case improved the function of the anterior cingulate and prefrontal cortices, thereby activating the descending inhibitory system and improving central sensitization and hyperalgesia.

Originally, two quantitative sensory tests, static quantitative sensory testing (QST) (pressure pain thresholds, thermal stimuli, and cutaneous mechanical stimuli) and dynamic QST (temporal summation and conditioning pain modulation), were proposed as methods for evaluating central sensitization (56). However, implementing temporal summation and conditioning pain modulation in a clinical or research setting is challenging due to the expensive equipment needed and the time-consuming measurement process. QST is a method to indirectly study central sensitization, which is assumed to underlie the phenomenon of pain hypersensitivity detectable by QST. However, QST does not directly measure central sensitization. In clinical practice, patients with NP can use a self-administered questionnaire like the CSI-9 for evaluation, as done in this study. Caution is advised when evaluating central sensitization with the CSI-9 alone, as it includes symptoms like morning fatigue, muscle pain, general pain, headache, difficulty concentrating, and difficulty recalling, and assesses the degree of “central sensitization syndrome,” not central sensitization itself.

This study has four limitations. First, MP usage should be avoided in patients with severe anxiety or agitation (57). As anxiety and agitation, although not extremely severe, were observed in this case, MP should be administered more cautiously. Second, caution should be taken when combining MP and VFX due to their potential to raise heart rate and blood pressure (58) or cause serotonin syndrome (59). This can lead to an increased risk of adverse cardiovascular effects. However, no significant changes in heart rate, blood pressure, or cardiovascular issues were observed in this patient. There were no signs of serotonin syndrome, such as a decline in mental state, hyperthermia, or increased autonomic and neuromuscular activity. Third, the patient's MPI shifted from the DYS type to the ID type, indicating that although the symbiotic relationship with the husband's overprotection has ended, the patient is still experiencing interpersonal difficulties. Hence, it may be necessary to provide not only pharmacotherapy but also further psychological approaches like self-assertion training in multidisciplinary pain medicine, primarily targeting the AC type (2). Fourth, the patient took 823 days of treatment to resume work, suggesting that the intervention in this study may not have directly improved NP, and the patient may have naturally recovered with time. However, the research design of the case report does not allow for the establishment of a counterfactual, meaning a situation where the intervention did not occur. Consequently, it is impossible to determine the causal relationship between treatment and improvement. A future randomized trial is needed to determine the causal relationship between the intervention and improvement observed in this study. It is worth noting that the patient did not receive any NP-related treatment at any other medical institution after visiting our clinic. The above limitations should be noted when generalizing the findings of this case.

This case report highlights the detailed process of evaluating, diagnosing, and treating an abdominal NP with ADHD, ASD, and TRD. Furthermore, it indicates that ADHD medications and the antidepressant VFX can improve chronic pain, family relationships, central sensitization, and cerebral blood flow. NP accompanied by comorbid ADHD can be expected to improve with ADHD medications; however, when depression is also present, prioritizing treatment with antidepressants may be necessary. Therefore, VFX may also be a useful antidepressant for patients with comorbid ADHD and ASD. However, this is the only case report describing the treatment course for abdominal NP in an adult with comorbid ADHD, ASD, or depression. Further research is required to establish a link between these disorders and their treatment.

## 4 Patient perspective

She said, “Through this treatment, I have not only improved my pain, but I have also gained a better understanding of my developmental characteristics, and I now have a better understanding of why I have had difficulties with interpersonal relationships so far. In the future, I would like to work on managing my behavior as well as my medications.”

## Data Availability

The original contributions presented in the study are included in the article further inquiries can be directed to the corresponding author.
